# Study Design Considerations for the Evaluation of Pancreatic Enzyme Replacement Therapy (PERT) Products in People with Cystic Fibrosis: An Update

**DOI:** 10.1016/j.clinthera.2026.04.021

**Published:** 2026-05-18

**Authors:** Michael W. Konstan, Mitchell L. Ramsey, Meghana Sathe, A. Jay Freeman, Steven D. Freedman, Sarah J. Schwarzenberg, Colin O’Rourke, Frankline M. Onchiri, Linda Setiawan, Joseph M. Pilewski, Nicole Mayer-Hamblett

**Affiliations:** 1 Department of Pediatrics, Case Western Reserve University School of Medicine/Rainbow Babies and Children’s Hospital, Cleveland, Ohio; 2 Division of Gastroenterology, Hepatology, and Nutrition, The Ohio State University Wexner Medical Center, Columbus, Ohio; 3 Division of Pediatric Gastroenterology, Hepatology and Nutrition, University Texas Southwestern/Children’s Health, Dallas, Texas; 4 Division of Gastroenterology, Hepatology, and Nutrition Nationwide Children’s Hospital, Columbus, Ohio; 5 Division of Gastroenterology, Beth Israel Deaconess Medical Center, Boston, Massachusetts; 6 Division of Pediatric Gastroenterology, Hepatology, & Nutrition, University of Minnesota, Minnesota; 7 Center for Respiratory Biology and Therapeutics, Seattle Children’s Research Institute, Seattle, Washington; 8 Department of Medicine, Division of Pulmonary, Allergy, Critical Care and Sleep Medicine, University of Pittsburgh, Pittsburgh, Pennsylvania; 9 Department of Pediatrics and Department of Biostatistics, University of Washington, Seattle, Washington

**Keywords:** Cystic fibrosis, Study design, Exocrine pancreatic insufficiency, Pancreatic enzyme replacement therapy

## Abstract

**Purpose:**

In 2013, the Cystic Fibrosis Foundation (CFF) published recommendations regarding study design considerations for the evaluation of porcine derived pancreatic exocrine replacement therapy (PERT) products in people with CF and exocrine pancreatic insufficiency (EPI). The purpose of this report is to provide an updated guidance with an emphasis on the design and conduct of non-inferiority (NI) trials of non-porcine derived PERT products.

**Methods:**

A CFF PERT Working Group comprised of gastroenterologists, statisticians, and authors of the 2013 publication was established to produce an updated guidance document based on their expertise in clinical trial design and outcome measures in people with CF and EPI.

**Findings:**

For the evaluation of non-porcine PERT products, we recommend a parallel withdrawal design to demonstrate the non-inferiority of the investigational product to an active comparator (an approved porcine PERT product). Sample size calculations and other statistical considerations for NI studies are provided. To determine EPI eligibility, we recommend fecal elastase-1 (FE-1) testing rather than an off-PERT period. For assessing efficacy, we recommend including a substrate absorption challenge test (SACT) for comparison to the currently accepted coefficient of fat absorption (CFA) to further advance an alternative, more feasible primary outcome measure. A patient reported outcome measure (PROM) specific to CF-EPI and acceptable to regulatory agencies needs to be defined, several are under investigation.

**Implications:**

Observations and recommendations provided in this updated report should benefit clinical investigators, drug developers, and regulatory agencies as they consider the issues and nuances of evaluating PERT products for cystic fibrosis.

## Background

Cystic fibrosis (CF) affects nearly 40,000 children and adults in the United States. In people with CF (pwCF), mutations in the cystic fibrosis transmembrane conductance regulator (*CFTR*) gene result in a buildup of thickened secretions in multiple organ systems, leading to lung damage, life-threatening infections, and other complications, including exocrine pancreatic insufficiency (EPI). PERT is indicated and prescribed for the treatment of EPI in approximately 85% of pwCF and is often regarded as a lifesaving therapy. Prior to the introduction of PERT, pwCF, including young babies, often died from malnutrition associated with EPI.

Although CFTR modulators, which directly target the underlying defects in the processing, trafficking, and function of the CFTR protein, have drastically altered the CF therapeutic landscape and the lives of up to 90% of pwCF who are eligible for and able to tolerate these drugs, the vast majority of pwCF still suffer from significant EPI and take PERT products with every meal to avoid malnutrition and other complications associated with EPI. Current PERT products (pancrelipase) are manufactured from porcine pancreatic extracts (pancreatin), but concern over the supply availability of pig pancreases for pancreatin, the susceptibility of PERT production to zoonotic infection, and additional community desire for porcine-free options has prompted clinical development programs for PERT products bioengineered from nonanimal sources. The CF Foundation provided recommendations in 2013^1^ for clinical trial design and outcome measures that were consistent with a 2006 FDA Guidance for Industry.^2^ Both were aimed at enabling New Drug Application (NDA) submissions mostly for existing porcine PERT products (termed pancrelipase) and recommended trial designs that incorporated short study periods off PERT or on placebo.

Neither of these documents addressed study considerations for non-porcine PERT products nor the need and analysis of rapid disintegrating PERT formulations, the latter needed for infants or other individuals unable to swallow large capsules. It is notable that in 2020 the FDA with-drew the Guidance to Industry,^3^ and to our knowledge no subsequent guidance has been released. In this report, we provide recommendations for consideration of an updated regulatory guidance for study designs evaluating investigational PERT products in pwCF.

### Study Design Considerations

#### Cross-Over and Parallel Designs

Our 2013 report discussed the details surrounding the three main study designs recommended by the FDA guidance to industry using placebo as the comparator and CFA as the efficacy measure: cross-over, randomized withdrawal, and parallel.^1^ In the cross-over design, subjects are randomized 1:1 in a cross over fashion to investigational PERT-placebo or placebo-investigational PERT. CFA is determined during both periods, and subjects serve as their own control. This design was the most frequently used design in NDA enabling studies in subjects with CF that led to FDA marketing approval of the pancrelipase products Creon,^4^ Zenpep,^5^ Ultresa,^6^ and Pertzye.^7^ The attractiveness of the cross-over design lies in its simplicity and need for fewer subjects to demonstrate efficacy compared to parallel designs. However, many assumptions are made with the cross-over design, and bias may not be accounted for in analyses. These assumptions and biases were previously discussed in detail in our 2013 report.

In the randomized withdrawal design (referred to as parallel withdrawal in our 2013 report and in this updated report), several scenarios have been used. Among these include a study where all eligible subjects undergo an open-label run-in period with an investigational PERT, followed by a baseline CFA on the investigational PERT. Subjects achieving a CFA >80% on the investigational PERT are then randomized to continue the investigational PERT or switch to placebo. A second CFA is obtained at the end of a double-blind treatment period for comparison to the baseline CFA. This design was used by an FDA NDA enabling study that led to the marketing approval of the pancrelipase product, Pancreaze.^8^ A second scenario for a placebo-controlled randomized withdrawal design is one in which eligible subjects undergo a baseline CFA while off their clinically prescribed PERT, followed by open label treatment with the investigational PERT. Subjects with baseline CFA <80% are then randomized to either continue the investigational PERT product or switch to placebo, followed by a second CFA for comparison to the baseline CFA. An example of this approach was a phase 3 parallel-group, randomized-withdrawal, double-blind placebo-controlled trial of a non-porcine investigational PERT product, liprotamase.^9^ This approach is less biased and more representative of the CF population treated with PERT but requires a longer period off PERT. Yet another variation of a parallel withdrawal design was used by a more recent trial of liprotamase, evaluating the non-inferiority of the investigational PERT to an active comparator (rather than superiority to placebo).^10^ Eligible subjects undergo an open-label run-in period with a marketed pancrelipase, followed by a baseline CFA on that pancrelipase. Subjects achieving a CFA >80% are then randomized to continue a pancrelipase or (withdraw) to the investigational PERT, followed by a second CFA for comparison to baseline. This non-inferiority active comparator design was not described in the 2006 FDA guidance or in our 2013 report but discussed in greater detail below.

In the placebo-controlled parallel design (referred to as parallel reintroduction in our 2013 report), a baseline CFA is obtained off PERT, and those with CFA <80% off PERT are randomized to receive either the investigational PERT or placebo. A second CFA is obtained at the end of the double-blind treatment period for comparison to the baseline CFA. To our knowledge, this design has not been used in clinical trials of PERT in CF.

#### Placebo vs Active Comparator

The most robust way to determine short-term efficacy of a PERT product is to demonstrate superiority to placebo, using CFA as the efficacy outcome. Our 2013 report provided sample size calculations for cross-over, parallel withdrawal, and parallel reintroduction designs using placebo as comparator to a PERT product.^1^ We emphasized that study designs using placebo should minimize the number of subjects receiving placebo and that the duration of placebo or off PERT periods should be as short as possible to lessen the risk to subjects. Compared to parallel designs, we showed that a cross-over design requires half the number of subjects to demonstrate efficacy. Thus, it is not surprising that most NDA-enabling studies of approved pancrelipase products employed a cross-over design,^4–7^ despite the many inherent assumptions and biases with this design. As demonstrated in our 2013 report, although parallel withdrawal and parallel reintroduction designs have similar sample size requirements, the reintroduction design requires three-times as many courses off PERT or on placebo. It is not surprising that the reintroduction design was not used for any of the NDA-enabling studies of approved pancrelipases.

For the NDA enabling placebo-controlled trials cited in this report, study discontinuations due to adverse effects occurred more frequently during off-PERT or placebo periods (abdominal pain, distal intestinal obstruction syndrome, weight loss), sometimes necessitating additional subject recruitment to complete study enrollment. It has become increasingly difficult to recruit subjects for participation in “placebo” PERT trials, as many pwCF are reluctant to discontinue PERT for even 1 day fearing anticipated increase in signs and symptoms associated with untreated EPI. Additionally, IRBs have become more cognizant of safety issues related to trials that use placebo and/or off PERT periods, as they consider study risk/benefit.

Due to the above issues related to placebo use in PERT trials, we strongly recommend and prefer study designs that use active comparators (currently marketed PERT products) rather than placebo. We recognize that this may increase study sample size regardless of study design chosen (cross-over or parallel), but using an active comparator most importantly avoids the safety issues associated with use of placebo while minimizing recruitment issues due to hesitancy of pwCF and their care providers to participate in trials with risks associated with stopping standard of care PERT. Moreover, using an active comparator will allow for longer duration trials on study drug compared to placebo studies. Unlike placebo-controlled trials, double-blind masking of trials that use active comparators is not possible given that a PERT product as an active comparator comes in a variety of doses per capsule (eg, 8,000, 16,000, 24,0000 lipase units), and a large range of capsules per meal (eg, 2, 3, 6) are prescribed based on the number required to control signs and symptoms of EPI. Thus, the active comparator cannot be masked to appear like the investigational product. Because masking is not possible, active comparator trials of investigational PERT products (eg, liprotamase) have resorted to an open-label blinded assessor design.^10^

#### Non-inferiority Trial Design for Evaluating Non-porcine PERT Products

All the FDA NDA-enabling studies of approved porcine PERT products followed the 2006 FDA guidance to industry and demonstrated superiority of porcine PERT products compared to placebo in short duration efficacy studies. Concern over the supply availability of pig pancreases for porcine PERT products, the susceptibility of PERT production to zoonotic infection, and additional community desire for porcine-free options has prompted clinical development programs for PERT products bioengineered from non-animal sources.^9–11^ Neither the 2006 FDA guidance to industry nor our previous report addressed study design considerations for non-porcine PERT products. Regulatory approval for non-porcine PERT products would likely require longer-duration efficacy and safety trials comparing the investigational product to a currently marketed porcine PERT product (pancrelipase) rather than placebo and likely show non-inferiority to the active comparator. A non-inferiority trial aims to show for the designated primary outcome that the investigational product (eg, a non-porcine PERT formulation) is not clinically meaningfully worse than the comparator (eg, a currently marketed porcine PERT product). A predefined non-inferiority margin represents the largest extent to which the investigational product’s effectiveness can be lower than that of the comparator while still being considered clinically meaningful.

Several development programs of investigational non-porcine PERT products have used, or plan to use, a parallel withdrawal design evaluating the non-inferiority of an investigational PERT to an active comparator. One such study was a phase 3 randomized, open-label, assessor-blind, parallel-group non-inferiority trial comparing liprotamase, a biotechnology-derived non-porcine PERT product containing highly purified lipase, protease and amylase, to a porcine PERT product (the pancrelipase Pancreaze).^10^ In this trial, 183 subjects ≥ age 7 years with CF associated EPI were screened and underwent a run-in period with a marketed pancrelipase (Pancreaze excluded) for at least 4 weeks, after which a baseline CFA was determined. 129 subjects who met screening criteria including a CFA >80% on pancrelipase were then randomized 1:1 to receive either liprotamase or Pancreaze (as the active comparator pancrelipase), with doses matched to pre-randomization lipase doses. A second CFA was obtained at the end of an 8-week primary treatment period, followed by a 12-week extension period on liprotamase or Pancreaze. The primary endpoint was the between-group difference in mean change from baseline in CFA after the primary treatment period. Non-inferiority was assessed using a pre-specified margin of −15%, with liprotamase considered non-inferior if the lower bound of the 95% confidence interval (CI) for the difference did not fall below −15%. The study did not demonstrate non-inferiority of liprotamase to pancrelipase: the lower bound of the 95% CI was −16%, just beyond the prespecific margin. Key secondary endpoints included the difference in CNA and signs and symptoms of malabsorption. This trial provides useful context for discussing how non-inferiority margins are selected in regulatory settings.

### Statistical Considerations for Designing Non-inferiority Trials

#### Defining an Objective Non-inferiority Margin.

A non-inferiority margin represents the largest loss of efficacy that would still be considered clinically acceptable when adopting a new treatment in place of an active comparator.^12,13^ It defines the threshold below which the new treatment would be considered unacceptably worse when compared to the treatment currently in use. The −15% margin used in the liprotamase vs. Pancreaze trial is an example of such a threshold.^10^ In the 2016 FDA guidance for industry on non-inferiority trial design, one recommended method for defining this margin is the fixed-margin approach, which relies on data from placebo-controlled trials of the active comparator.^13^ Historical studies used for this purpose should be sufficiently similar to the proposed trial in their design, conduct, patient population, and endpoints. If multiple qualifying studies exist, their results may be synthesized using a meta-analytic approach to estimate the active comparator’s effect versus placebo. The lower bound of the 95% confidence interval around that estimate is then taken as a conservative measure of the smallest plausible benefit. The non-inferiority margin is selected so that the investigational treatment preserves a clinically meaningful portion of that benefit—typically at least 50 percent, as noted in FDA guidance. However, the exact fraction is context-dependent: for highly effective comparators, a tighter margin may be warranted to avoid an unacceptable loss of efficacy, while in other settings regulators may agree that a larger margin is appropriate. Thus, the 50% preservation rule should be viewed as a starting point for discussion rather than a fixed requirement. Once the margin is established, the trial is designed to test whether the observed difference between treatments meets this threshold; non-inferiority is concluded when the lower bound of the 95% confidence interval for the treatment difference lies above the prespecified margin.

Although less commonly employed in regulatory practice, the FDA guidance also describes an alternative framework: the *synthesis method* (sometimes referred to as the *putative placebo* approach).^12–14^ This method is applied after the non-inferiority trial is complete and uses two sources of information: the historical effect of the active comparator versus placebo and the observed effect of the test treatment versus the active comparator in the current trial. These two estimates are combined statistically to estimate how the test treatment might have performed had it been compared directly to placebo in a separate trial. To conclude non-inferiority under this method, a target level of preserved benefit must still be prespecified—for example, that the new treatment should retain at least 50% of the benefit previously shown by the active comparator. As with the fixed-margin approach, the historical estimate is typically based on the lower bound of the 95% confidence interval to provide a conservative benchmark. While this method avoids setting a fixed margin on the measurement scale in advance, it still depends on a clinical judgment and regulatory agreement regarding what constitutes an acceptable loss of efficacy.

#### Sample Size Considerations for Non-inferiority Trials.

Once a non-inferiority margin has been selected, other key design parameters—such as outcome variability and expected treatment effect—directly influence the number of participants required. For example, in the liprotamase vs. Pancreaze trial, the planned sample size was based on a non-inferiority margin of 15%, an assumed standard deviation of 18% for change in CFA, and a 4% treatment effect difference favoring the active comparator.^10^ These assumptions led to a target enrollment of 126 participants (63 per group) to achieve 92% power at a one-sided significance level of 0.025.

**Figure 1.**
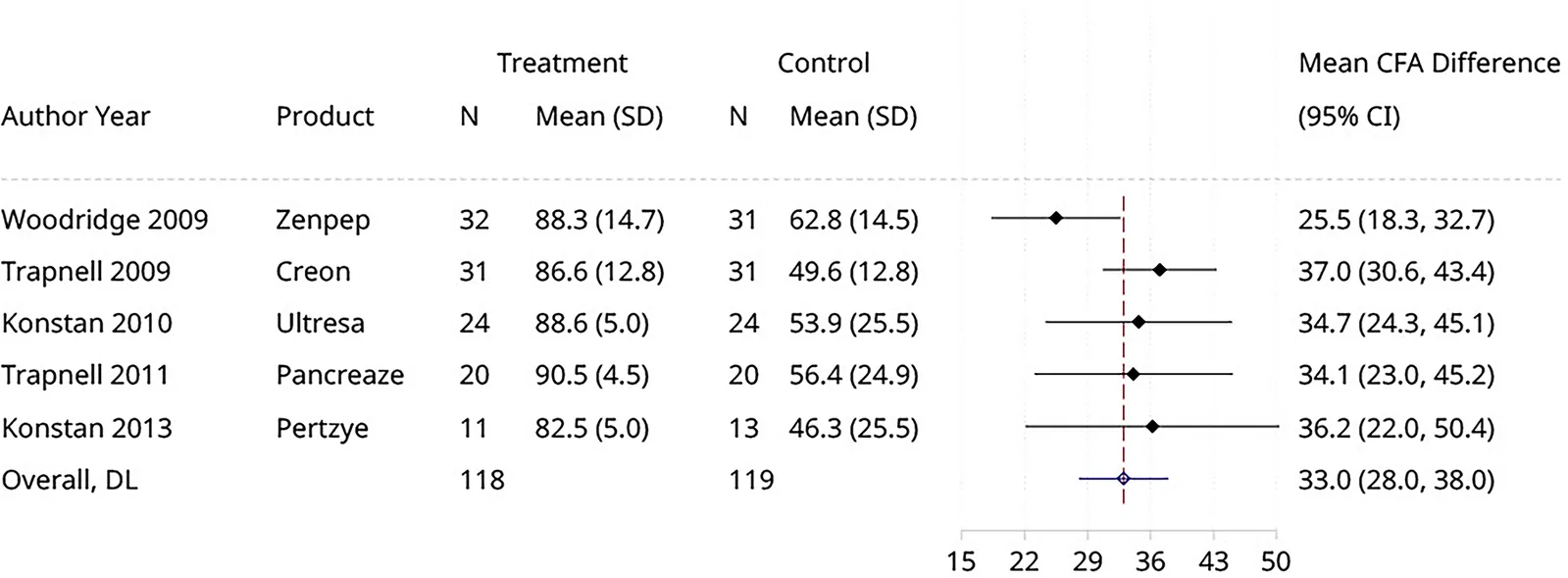
Results of meta-analysis for 5 placebo-controlled studies of PERT products. Under the fixed-margin approach, planning a study of an investigational PERT against an active comparator might result in the meta-analysis shown here. This example pools 5 potentially relevant studies to estimate the effect of the active comparator against placebo to be a 33% (95% CI 28% to 38%) increase in CFA. Based on the lower bound of this 95% confidence interval, to preserve at least 50% of the active comparator’s effects against placebo, setting a non-inferiority margin of at most 14% would be necessary to ensure that the investigational treatment preserves at least 50% of the active comparator’s effect against placebo.

**Figure 2.**
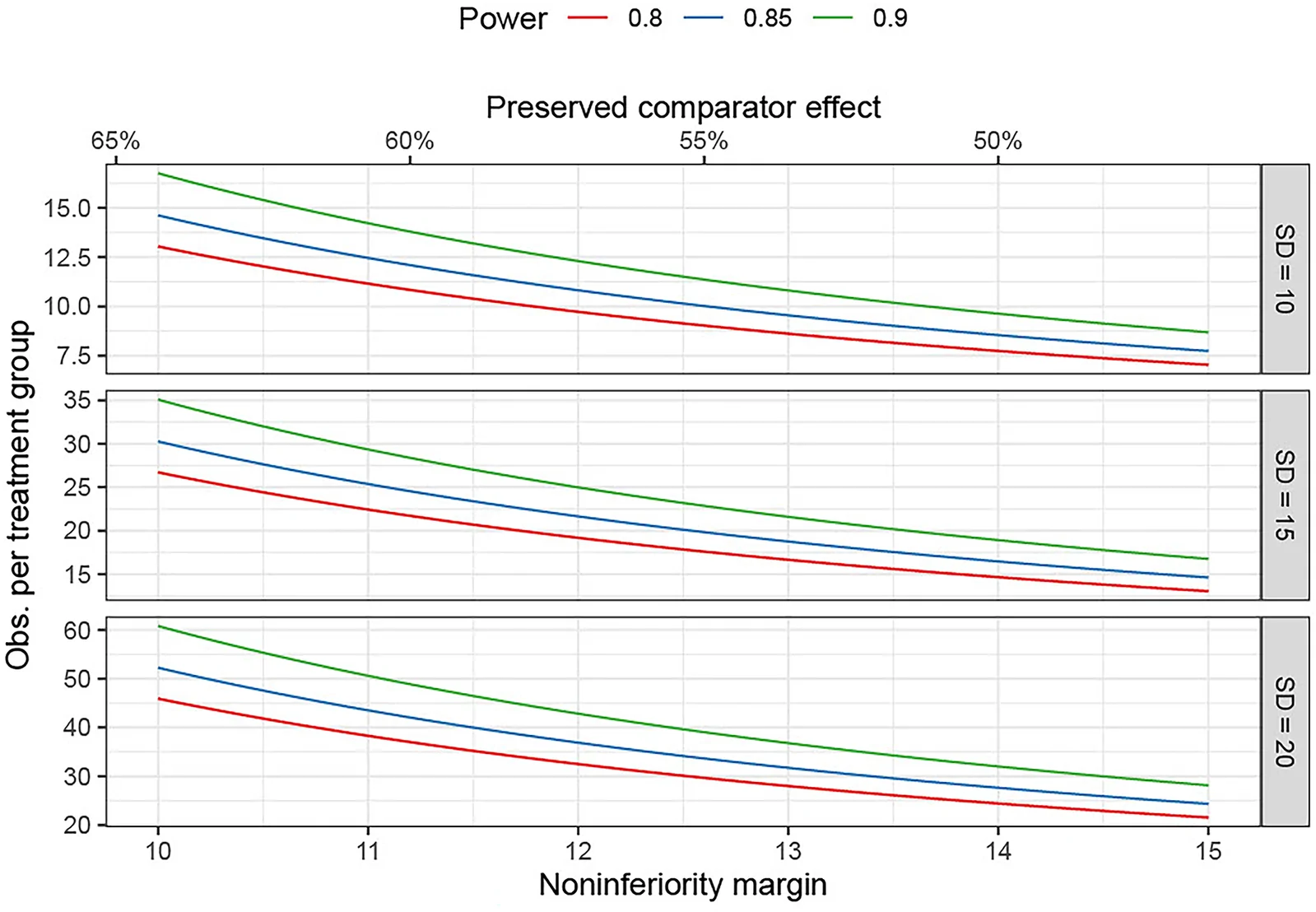
Total sample size required for a cross-over trial as a function of the chosen non-inferiority margin. Separate panels represent CFA standard deviations of 10%, 15%, and 20%, and curves show required sample sizes for achieving 80%, 85%, and 90% power. Calculations assume a non-inferiority test using a paired t-test, equal standard deviations across treatments, no true difference in mean CFA levels between PERT treatments. A within-subject correlation of 0.3. The preserved comparator effect value assumes a lower bound of 28% on the effect of the active comparator against placebo.

**Figure 3.**
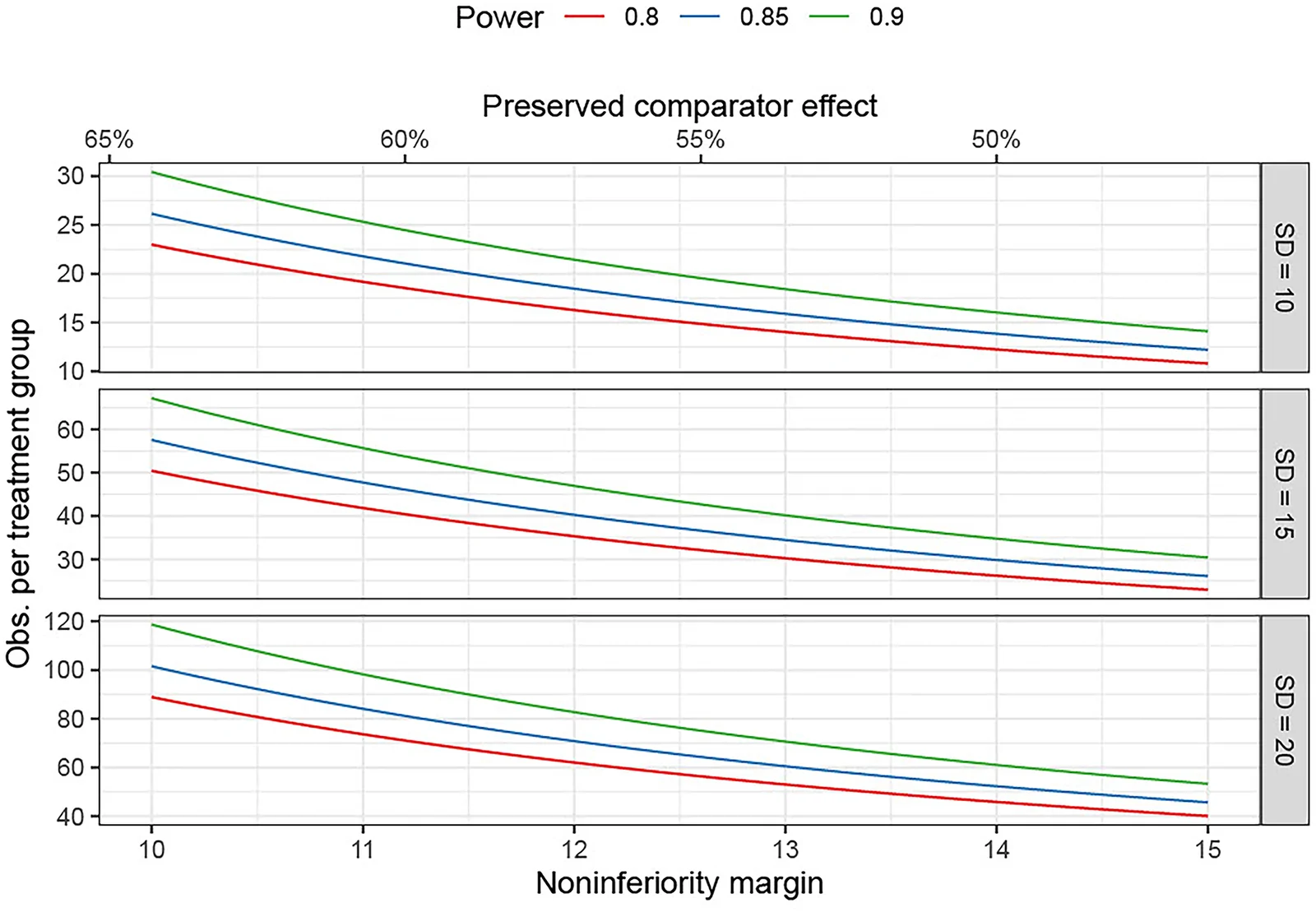
Total sample size required for a parallel withdrawal trial as a function of the chosen non-inferiority margin. Separate panels represent CFA standard deviations of 10%, 15%, and 20%, and curves show required sample sizes for achieving 80%, 85%, and 90% power. Calculations assume a non-inferiority test using a two-sample t-test comparing change in CFA from baseline, equal standard deviations across treatments, and no true difference in mean CFA levels between PERT treatments. A correlation of 0.3 was assumed between baseline and treatment CFA measurements made within a given subject. The preserved comparator effect value assumes a lower bound of 28% on the effect of the active comparator against placebo.

To understand how different design choices affect sample size requirements, we conducted power calculations across a range of plausible scenarios for non-porcine PERT trials. Among the studies reviewed in Figure 1, the standard deviation of CFA measurements under active treatment ranged from approximately 5% to 15%. We also reviewed published studies to estimate the correlation between two CFA values measured on the same subject. These ranged from 0.2 to 0.4, and we selected 0.3 for our calculations. For the parallel withdrawal design, we assumed equal group sizes. Figures 2 and 3 summarize sample sizes required under these assumptions. Figure 2 presents results for a two-sequence, two-period (2 × 2) cross-over design; Figure 3 shows results for a parallel withdrawal design. In both cases, power was assessed using a paired t-test and two-sample t-test, respectively, and assumes no difference in the true means by treatment group. Alternative assumptions about true treatment effects could be made depending on the context of a particular study. Each figure includes panels for three raw CFA standard deviations spanning the range observed in the literature. The involvement of a statistician is important to decide whether these assumptions are appropriate for a particular study, or whether new calculations should be done based on an alternative set of assumptions.

**Figure 4.**
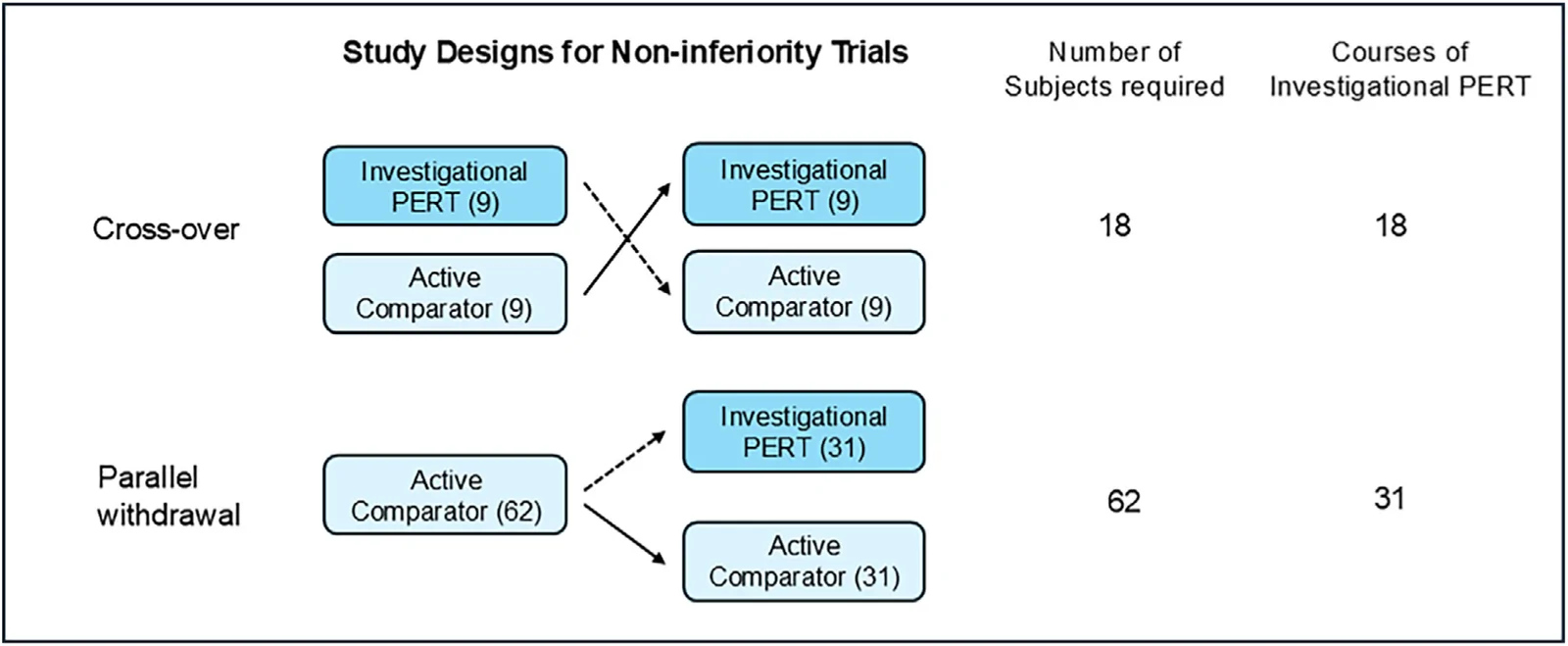
Illustration of cross-over and parallel withdrawal study designs for testing a non-inferiority hypothesis comparing an investigational PERT to an active comparator. Sample sizes reflect calculations for 90% power using a non-inferiority margin of 15%, a CFA standard deviation of 15%, and a within-subject correlation of 0.3. In the cross-over design, treatment groups are compared using mean within-subject differences in CFA. In the parallel withdrawal design, the comparison is based on mean changes from baseline between treatment groups.

For illustration, consider a scenario in which CFA variability is assumed to have a standard deviation of 15% and the non-inferiority margin is set at 15%. To achieve 90% power under these assumptions, a parallel-groups trial would require at least 31 participants randomized to each treatment. By contrast, a cross-over design under the same conditions would require only 18 total participants to achieve the same level of power. Figure 4 illustrates these results and designs.

### Use of Fecal Elastase as an Enrollment and/or Stratification Criterion for Defining EPI Disease Severity

Fecal elastase-1 (FE-1) testing is used clinically to diagnose EPI disease severity in pwCF, and is considered superior to fecal lipase testing.^15^ Several studies have evaluated FE-1 levels in pwCF to diagnose EPI without resorting to direct measures of pancreatic function or 72-hour stool collection for fecal fat. In 78 subjects with CF age 6 to 33 years with EPI based on a 72-hour stool fat balance (CFA) study, FE-1 values ranged from 0 to 107 ug/g of stool, with a mean of 15.8 and a median of 6.5.^15^ In 1215 subjects with CF age >1 month (mean age 13.5 years) irrespective of a diagnosis of EPI, FE-1 values ranged from 0 to 867 ug/g of stool, with a median of 0 ug/g stool. 86.9% had an FE-1 < 100, 1.5% had an FE-1 between 100 and 200, and 11.5% had values >200.^16^ Of those with values <200, 98% were previously prescribed PERT, and thus diagnosed with EPI based on clinical findings. A subset of the subjects with FE-1 values > 200 underwent a fecal fat balance test and were found to have CFAs in the normal range (CFA >93%).^16^ Based on these and previous studies, FE-1 <200 ug/g of stool is consistent with a diagnosis of EPI, with levels <100, especially with signs or symptoms of steatorrhea and/or micronutrient deficiency (eg, low serum vitamin A or E levels) consistent with more severe EPI.^17^ It is imperative that FE-1 testing be done on a formed or semiformed stool; loose or watery stools provide spurious lower results due to dilution.^18^ Clinical trials of PERT in CF often screen for or use history of FE-1 < 100 ug/g of stool as an inclusion criterion for participation.^5–10,19^ Some studies have required FE-1 < 50 to enhance for a study population with more severe EPI,^4,11,20^ while a study in infants and young children with CF was even more stringent requiring an FE-1 < 15.^21^ Although some studies have used a “low” off PERT CFA (eg, <80% or <70%) with or without FE-1 criteria for defining EPI as an inclusion criteria,^4,9,20,22^ there are significant shortcomings in using off PERT CFA for this purpose. These shortcomings are discussed in the online supplement. Thus, we recommend FE-1 testing as the preferred screening method for defining EPI disease severity, and propose an FE-1 of <50 micrograms/gram of stool as an inclusion criterion for participation in PERT trials

### Concomitant Medications

Some studies employed very strict exclusion criteria regarding prohibiting the use of any medication during the trial that had the potential to affect gastric motility or pH, including proton pump inhibitors (PPI), histamine-2 blockers, motility agents, buffering agents, laxatives including mineral oil, castor oil, and polyethylene glycol, agents for gastric ulcers, synthetic fat substitutes, and fat-blocking nutritional supplements^5^, while others were less strict or specific regarding excluding these medications.^4,6,8,23^ Some studies that allowed gastric acid suppressants randomized subjects at enrollment based on their use.^9,10^ In the previously cited study of liprotamase compared to pancrelipase, concomitant gastric acid suppressant use was associated with higher CFA with liprotamase but not pancrelipase, leading the investigators to speculate that gastric acid suppressants may improve the efficacy of this formulation of liprotamase in future trials.^10^ A recent randomized cross-over trial specifically designed to determine if PPI improved the efficacy of various pancrelipase products also failed to show a higher CFA with concomitant PPI use.^24^ For studies of non-porcine PERT products, consideration should be given for randomization on enrollment based on gastric acid suppressant use.

CFTR modulators are another category of medications that might affect the efficacy of PERT. A scoping review cited several studies that reported improvement in exocrine pancreatic function in young children after CFTR modulator treatment based on increases in FE-1, decreases in serum pancreatic enzyme levels, and lowering of PERT dosing.^25^ A more recent report revealed 12 of 43 children age 2 to 6 years with EPI became pancreatic sufficient (FE-1 > 200) after 6 months of elexacaftor/tezacaftor/ivacaftor (ETI)^26^ while another observational study revealed no change in FE-1 in 45 older children and adults (age ≥12 years) after 6 months of ETI.^27^ These findings suggest that PERT trials in young children should consider stratifying at randomization based on CFTR modulator use and minimize CFTR modulator dose adjustment.

### Study Design Considerations for Young Children

Although children make up a substantial number of pwCF requiring PERT to treat EPI and thus optimize their nutritional status, including infants and young children in clinical trials of PERT is challenging. Given that the outcome measure currently accepted by regulatory agencies to assess PERT efficacy is the CFA, the 72-hour controlled diet and marker-to-marker stool collection procedures for determining CFA are difficult to perform in children not old enough to adhere to and consume a prescribed (palatable) high-fat diet and to save stool from each bowel movement to assess for the presence of the dye marker. Although there are examples in the literature of studies using CFA in infants with CF,^21,22,28^ adherence to a controlled diet and obtaining stool from toddlers is difficult and likely more unreliable compared to older subjects. In fact, the NDA enabling studies of PERT cited earlier excluded children younger than age 7 years due to the challenges associated with CFA procedures. Moreover, most enzyme formulations of PERT products consist of capsules containing enteric coated microspheres or microtablets. Administering these PERT products to infants and toddlers too young to swallow capsules requires sprinkling the capsule contents into apple-sauce or some other approved food source, making PERT dosing and study blinding challenging. Finally, close attention to the occurrence of adverse events, including weight loss or poor weight gain and other signs and symptoms of GI intolerance must be judiciously performed throughout any PERT trial including infants and children as participants. Although we have already recommended against study designs that use off PERT periods or placebo (rather than active comparators), we emphatically recommend against their use in PERT studies that include infants and young children.

### Outcome Measures

#### Biologic Outcome Measures

EPI is caused by a reduction in pancreas exocrine function to a degree that there is evidence of fat malabsorption.^17^ Measures of exocrine function are useful for diagnosis of EPI but not for monitoring the efficacy of PERT, as noted above for FE-1. Efficacy measures must instead quantify absorption or malabsorption of nutrients. Typically, these measure fats or fat-soluble nutrients since these are more relevant to pancreatic function than carbohydrates or proteins which also rely to some extent on nonpancreatic enzymes. An ideal efficacy measure would demonstrate a dose-response relationship, produce reliable results at all stages of exocrine dysfunction, and limit the burden on research subjects so it can be measured serially and incorporated into clinical practice. Historically, the coefficient of fat absorption has been the standard PERT efficacy measure, but newer substrate absorption challenge tests (SACTs) have since been developed to better address the need for an optimal PERT efficacy outcome measure. CFA, SACTs, and other efficacy measures quantifying absorption or malabsorption of nutrients are presented in greater detail in the Supplement. Recently published systematic literature reviews provide even more detail regarding these outcome measures in PERT trials.^29,30^

#### Clinical and Patient Reported Outcome Measures

Clinical measures including body weight; stool weight, frequency and characteristics; and EPI-related symptoms like abdominal cramps and flatulence are commonly used as secondary efficacy measures in PERT studies. In addition to these clinical measures, patient reported outcomes measures (PROMs) for response to PERT should also be incorporated into studies of PERT products. Patient reported outcome measures (PROMs) for EPI include general gastroenterology PROMs which incorporate upper and lower gastrointestinal surveys, such as the Patient Assessment of Upper Gastrointestinal Disorders-Symptom Questionnaire (PAGI-SYM)^31^ and the Patient Assessment of Constipation Symptom and Quality of Life Questionnaires (PAC-SYM and PAC-QOL)^32,33^ and symptom indices for a single disease, such as the Cystic Fibrosis Abdomen Score (CFAbd-Score)^34^ or the Cystic Fibrosis Questionnaire-Revised (CFQ-R).^35,36^ PROMs tailored more specifically to EPI include the Pancreatic Exocrine Insufficiency Questionnaire (PEI-Q).^37–39^ At the present time, the PEI-Q seems to be the most complete PROM available. Clinical and patient reported outcome measures are discussed in greater detail in the Supplement.

### Safety Considerations

There are two major areas of safety to monitor during PERT studies: complications due to inadequate or absent enzyme activity from the investigational PERT product and complications due to toxicity associated with the PERT product. Both should be considered when assessing individual subjects and when developing overall stopping rules for a study. Questionnaires that monitor signs and symptoms of pancreatic insufficiency should be reviewed when submitted to identify subjects with significant abdominal pain, constipation, diarrhea, and other changes in GI signs and symptoms. Subjects with signs and symptoms that lead to distress or impact daily function should be considered for study withdrawal to allow them to resume their clinically prescribed PERT. More severe concerns would include weight loss and poor weight gain or growth (in children). Distal intestinal obstruction syndrome (DIOS) in pwCF is a complication generally due to insufficient PERT activity. The development of this condition should also be a reason for subject withdrawal. Although applicable only to studies of long duration, subjects experiencing a decline in fat-soluble vitamin levels without a change in vitamin supplementation should also be considered for study withdrawal.

Standard laboratory safety measures include monitoring electrolytes, blood urea nitrogen, liver enzymes (alanine aminotransferase, aspartate aminotransferase, gamma-glutamyl transferase) and complete blood count.^1^ These laboratory studies may vary in subjects for reasons unrelated to an investigational PERT product, but changes should increase concern for continued use. Because porcine-derived PERT products contain elevated levels of purines, monitoring subjects for hyperuricemia and uricosuria is an important safety measure.^23,40^

All studies should have a medical monitor available to study site personnel for emergent consultation, and Data Safety Monitoring Committees should be established for most studies. Interim reviews for safety and futility should be performed for longer duration studies.

## Summary

Key messages from this update to our 2013 report on study design considerations for the evaluation of PERT products in people with cystic fibrosis are presented in the Table 1. Observations and recommendations provided in this updated report should benefit clinical investigators, drug developers, and regulatory agencies as they consider the issues and nuances of evaluating PERT products for cystic fibrosis.

## Supplementary Material

Online Supplement

Supplementary material associated with this article can be found, in the online version, at doi:10.1016/j.clinthera.2026.04.021.

## Figures and Tables

**Table 1:** Key messages from the update on study design considerations for the evaluation of pancreatic enzyme replacement therapy (PERT) products in people with cystic fibrosis.

Study Design Considerations
For the evaluation of currently marketed porcine PERT products undergoing changes in formulation or manufacturing processes, previously described study designs (cross-over and parallel) to demonstrate efficacy over a short duration (days) remain appropriate.
For the evaluation of investigational non-porcine PERT products, we recommend a parallel withdrawal design to demonstrate the non-inferiority of the investigational product to a currently marketed PERT product over a longer duration (weeks). Longer duration allows for evaluation of safety in addition to efficacy.
Although current porcine PERT products gained marketing approval by demonstrating superiority to placebo in short duration studies using a cross-over or parallel design, conducting placebo-controlled trials of investigational PERT products is challenging due to safety concerns (increased gastrointestinal related adverse events on placebo) and subject recruitment and retention (subject discontinuations are greater for placebo vs PERT product). Moreover, recent placebo-controlled studies and even studies with periods off PERT to demonstrate baseline level of pancreatic insufficiency or with wash-out periods off PERT have had trouble gaining human subjects’ approval by Institutional Review Boards due to an unacceptable risk/benefit ratio. Thus, we recommend active comparator over placebo-controlled trials.
**Screening and Efficacy Measures**
We recommend fecal elastase-1 (FE-1) testing rather than off-enzyme coefficient of fat absorption (CFA) as the preferred screening method for defining EPI disease severity as an enrollment criterion for efficacy trials of PERT products. Unlike the 72-hour high-fat diet and marker-to-marker stool collection required for determining a CFA, FE-1 testing can be performed on a single stool specimen while on PERT and does not require a specified diet.
Due to the challenges associated with accurately determining CFA, consideration should be given to including alternatives to CFA as the primary outcome measure in clinical trials. We recommend that future trials of investigational PERT should include the O3-SACT measure for comparison to CFA results to further advance alternative, more feasible efficacy endpoints.
**Statistical Considerations**
Statistical considerations for a non-inferiority trial using CFA as the efficacy measure require an objectively defined non-inferiority margin. Once a non-inferiority margin has been selected, other key design parameters—such as outcome variability and expected treatment effect—directly influence the number of participants required. In the cross-over design, treatment groups are compared using mean within-subject differences in CFA. In the parallel withdrawal design, the comparison is based on mean changes from baseline between treatment groups.
**Patient Reported Outcomes**
The development and validation of a patient reported outcome measure (PROM) specific for CF-EPI is needed to meet regulatory requirements for inclusion as secondary outcomes in clinical trials. Several are under investigation, with the goal of defining a preferred PROM for trials of PERT products.
**Safety Considerations**
Safety monitoring for complications due to inadequate enzyme activity from the investigational PERT product and complications due to intolerance or toxicity is essential. A Data Safety Monitoring Committee should be established for most studies. Interim reviews for safety and futility should be performed for longer duration studies.
Special consideration should be given to studying PERT in children. For safety reasons, we strongly recommend against study designs that use off-PERT or placebo periods. Close monitoring for change in body weight and other signs and symptoms of gastrointestinal intolerance is paramount.
