# Supplementary material for: Study Design Considerations for the Evaluation of Pancreatic Enzyme Replacement Therapy (PERT) Products in People with Cystic Fibrosis: An Update
Source: Clin Ther. Author manuscript; Available in PMC 2026 Sep 2. (PMC13537623; doi:10.1016/j.clinthera.2026.04.021)
Supplement: Supplementary file 1 [file NIHMS2202510-supplement-Online_Supplement.pdf]

Konstan et al. Clinical Therapeutics, 2026; 48, 648-655

ONLINE SUPPLEMENT

Study design considerations for the evaluation of pancreatic enzyme replacement therapy (PERT) products in people with cystic fibrosis – An Update

BIOLOGIC OUTCOME MEASURES

Exocrine pancreatic insufficiency (EPI) is caused by a reduction in pancreas exocrine function to a degree that there is evidence of fat malabsorption [1]. Measures of exocrine function are useful for diagnosis but are not useful for monitoring the efficacy of PERT. For example, FE-1 is useful for diagnosing and staging the severity of EPI, but the value does not change after commencement of PERT. Efficacy measures must instead quantify absorption or malabsorption of nutrients. Typically, these will measure fats or fat-soluble nutrients since these are more relevant to pancreatic function than carbohydrates or proteins which also rely to some extent on non-pancreatic enzymes. An ideal efficacy measure would demonstrate a dose-response relationship, produce reliable results at all stages of exocrine dysfunction, and limit the burden on research subjects so it can be measured serially and incorporated into clinical practice. Historically, the coefficient of fat absorption has been the standard PERT efficacy measure, but newer substrate absorption challenge tests (SACTs) have since been developed to better address the need for an optimal PERT efficacy outcome measure. Efficacy measures quantifying absorption or malabsorption of nutrients are presented in greater detail in the following sections. Recently published systematic literature reviews provide even more detail regarding these outcome measures in PERT trials [2, 3].

Coefficient of fat absorption (CFA)

The currently accepted outcome measure for determining efficacy of a PERT product is the coefficient of fat absorption (CFA), which is determined from a 72-hour controlled high-fat diet and stool collection delineated by dye markers. Extensive details regarding the CFA procedures were provided in our 2013 report [4] and was used as the primary efficacy outcome measure in clinical trials in pwCF that led to FDA approval of the currently marketed porcine PERT products. Although regarded as the accepted efficacy measure, the CFA procedures involved are labor intensive, prone to error, and the results for

individuals are subject to inaccuracies. The procedure is best performed in a research setting, often in a hospital or other controlled setting, under the continuous supervision of research staff, using prepared meals with known fat, protein and carbohydrate content, and collecting stools based on the appearance of a dye marker taken at the beginning and the end of a 72-hour controlled diet. The stool collection period requires 5 to 7 days to complete, depending on the transit time of the dye markers. The accurate measure of fat in the collected stool (consisting of all stools after the first dye marker up to the stool that contains the 72-hour dye maker) is highly dependent on the research staff being able to accurately visualize for dye marker in stool from each bowel movement after administration of the first marker. Over-collecting stool results in a falsely lower CFA, while under-collecting stool results in a falsely higher CFA. Surprisingly, negative CFAs (meaning more fat appeared in stool than the amount ingested), physiologically impossible, have been observed in subjects with CF participating in PERT trials [5, 6]. High intra-subject variability in CFA in pwCF and EPI undergoing research-quality testing while off PERT has been reported [7], making CFA an unreliable screening and stratification tool. Moreover, a CFA in an individual subject obtained off PERT is not predictive of their CFA on PERT (e.g. a subject with a CFA of 20% off PERT can have a CFA of 80% on PERT, the same CFA on PERT for a subject with a CFA of 60% off PERT), as shown in a placebo-controlled cross-over study of pancrelipase (Creon) [8]. This finding prevents the use of CFA off PERT as a useful stratification factor for EPI severity. CFA has also been shown to be incapable of discriminating dose-response relationships [9]. Not only is hospitalization for research purposes expensive for trial sponsors and is often deprioritized by hospitals when available beds are limited, requiring hospitalization has become more difficult for subject recruitment, further impeding progress of PERT development programs. Moreover, recent survey results regarding PERT studies from "Community Voice", a Cystic Fibrosis Foundation sponsored group comprised of pwCF and parents of children with CF in the U.S., revealed that nearly half (46%) of respondents replied that they would not be willing and 25% were unsure regarding participation in a research study of a new PERT product that required two hospital stays for CFA testing [10]. Fecal fat absorption measured from sparse stool sampling during a high-fat diet has been proposed as more convenient and less complicated than the 72-hour CFA method but suggested only as a screening tool and not for clinical trial efficacy of a PERT product [11].

In addition to these logistical challenges, CFA has other important shortcomings that limit use in research studies and in clinical practice. The CFA is a whole gut "black box" method where fat input and fat output can be measured, but many important mechanisms of digestion and absorption are not specifically

measured [12, 13]. Several unmeasured confounders may affect the result, such as small bowel epithelial diseases, small bowel pH affecting PERT efficacy, timing of PERT ingestion in relation to the meal, and intestinal bacterial overgrowth. While non-pancreatic lipases perform only a small percentage of lipolysis in healthy individuals, these play a much larger role in individuals with severe EPI and limit the validity of CFA in this population [14]. These unmeasured confounders may also contribute to the significant test-retest variability described above [12].

In addition to the systematic literature reviews previously cited [2, 3], a recent evidence-based review of CFA to measure efficacy of PERT in people with CF highlights these and other issues and concluded that "developing suitable alternatives should be a priority" [12]. SACTs address this issue through dynamic/pharmacokinetic studies with serial measurements following an ingestion of a substrate that requires intraluminal lipolysis for absorption. Historically, several SACTs have been developed but are no longer widely used, such as the bentiromide-para-aminobenzoic acid (BT-PABA) test [15]. As described below, the most promising SACTs which could replace CFA as the primary outcome measure for future PERT studies measure serial blood levels following an ingestion of specific fatty acids which require intraluminal lipolysis to raise serum levels. These provide an assessment of real-time fat absorption and may be widely adopted as these metabolites can be measured in blood samples. Although the serial samples may lead to prolonged laboratory visits, this will likely be preferable to the dietary constraints and handling of stool required by the CFA. From the "Community Voice" survey cited above, 66% of respondents reported that they would likely or very likely participate in a PERT study that involved a single day in an outpatient research setting where serial blood tests would be used as an outcome. Only 29% of these same respondents expressed a willingness to participate in a PERT study that involved CFA testing that involved two hospital stays [10].

Measures of Fat Absorption using Substrate Absorption Challenge Tests (SACTs)

^{13}C-Mixed Triglyceride Breath Test

A SACT initially developed in the late 1980s involves an overnight fast, followed by consumption of a ^{13}C-labeled test meal [16]. Subjects collect breath samples at regular intervals for several hours afterwards—one standardized protocol measures breath samples at 15-minute intervals for 6 hours [17]. The appearance of $^{13}CO_2$ in breath signifies that digestion, absorption, and oxidation has occurred, so the serial breath samples provide a timeline of these processes and thus reflect a pharmacokinetic measure of fat absorption. However, this test also has several shortcomings, including significant test-retest

variability, low resolution for detecting dose-response changes, and limited availability due to the collection and analysis of breath testing [12].

Malabsorption Blood Test

The first SACT using serial blood testing was described in 2013 and is based upon differential absorption of pentadecanoic acid (a free fatty acid which can be readily absorbed) and triheptadecanoic acid (a triglyceride which requires hydrolysis by pancreatic lipase before heptadecanoic acid can be absorbed) [18]. After an overnight fast, a test meal containing these substrates is consumed and blood samples are drawn hourly for 8 hours to assess the ratio of heptadecanoic acid to pentadecanoic acid based on the absorption, defined as the area under the curve (AUC) for these two fatty acids.

In clinical studies, this Malabsorption Blood Test (MBT) has shown significant differences among healthy subjects at baseline and during orlistat treatment, among persons with cystic fibrosis (pwCF) at baseline and during treatment with PERT, and among subjects with chronic pancreatitis at baseline and during treatment with PERT [18-20]. Notably, the MBT and CFA were simultaneously assessed in 24 subjects with chronic pancreatitis before and after PERT and demonstrated significant improvement in the MBT but not in the CFA, suggesting that the MBT is a more sensitive efficacy measure [19]. Additionally, in a pilot study designed to assess the effects of proton pump inhibitors on fat absorption, the authors found no difference in MBT or CFA [21]. However, among the 13 subjects recruited, 9 completed two MBTs, while only three completed 2 CFAs. This suggests that subjects may find the MBT preferable to the CFA, which could facilitate larger sample sizes if MBT were selected as an outcome measure instead of the CFA.

Omega-3 Substrate Absorption Challenge Test (O3-SACT)

Another SACT utilizing serial blood testing, O3-SACT, uses a more widely available test meal—a fish oil supplement containing a pre-specified dose of the omega-3 fatty acids Docosahexaenoic (DHA) and Eicosapentaenoic Acids (EPA) [13]. These lipids normally have very low abundance in the blood, as they are the most difficult to hydrolyze by pancreatic lipase due to their number of double bonds. This allows for a pulse chase approach where a single large dose of DHA/EPA can be given orally and then be measured in plasma samples reflecting both lipolysis and intestinal absorption.

O3-SACT involves a 3-day run-in period with a diet low in omega 3-containing foods and no fish oil supplements. After an overnight fast, a test meal supplemented with a high dose of omega-3 DHA/EPA is administered, and blood drawn at serial intervals over 24 hours for assessment of plasma DHA and EPA

[22, 23]. The primary outcome measure is the AUC for total DHA plus EPA. In addition, these fatty acids in erythrocyte membranes can be measured after 3 months to assess fat absorption over the previous months [12, 13].

The O3-SACT was used as the primary efficacy measure for a study of a digestive enzyme cartridge designed to digest a fat-containing enteral feed. The cartridge contains a microbial-derived lipase and is attached in-line to an enteral feeding tube that connects to a gastrostomy port [22, 23]. In a multicenter double-blind cross-over trial, 33 subjects with CF were enrolled comparing the lipase-containing cartridge to a placebo cartridge. Plasma fatty acid levels improved significantly compared to placebo, into the ranges seen in healthy populations [23]. An open-label study found that omega 3 values improved at 30, 60, and 90 days of treatment with an immobilized lipase cartridge and an omega-3 enriched EN, compared to participant's baseline using standard PERT formulations [22]. Dose-response or comparison to CFA was not performed in these studies. After this study, validation of the O3-SACT was performed in an EPI pig model and showed that the test could distinguish between different doses of an orally delivered microbial-derived lipase [13]. Higher doses of the lipase with a test meal of omega-3 fatty acids resulted in higher plasma total fatty acids, and the effect on fatty acid absorption with the microbial-derived lipase was comparable to that obtained with porcine pancrelipase in this same model [13]. Another study in the same EPI pig model demonstrated a significant inverse relationship between 72-hour fecal fat content and serum levels of alpha linolenic acid, arachidonic acid, and docosahexaenoic acid [24], indicating a correlation between measured absorption in the blood and measured malabsorption in the stool. Most recently, the O3-SACT was used in a clinical trial of an investigational non-porcine PERT, ANG003 (consisting of microbially derived lipase, protease and amylase) [25]. In addition to omega 3, whey and potato starch were added to the test meal to assess for protein and carbohydrate absorption respectively. (Discussed below.) Absorption was assessed in 51 adult subjects with CF-related EPI once during an off-PERT period and again after randomization to a single dose level of four possible combinations of lipase, protease, and amylase. ANG003 lipase significantly improved DHA+EPA and total fat absorption in a dose dependent manner.

The O3-SACT has several advantageous features. First, as a blood-based SACT, the test is widely available. In addition, the test meal contains a commercially available omega 3 fatty acid supplement, which is also widely available. Second, short term pharmacokinetic studies have shown a dose-response relationship, and several fatty acids have a significant inverse relationship with total fecal fat content. These series of

studies suggest that the O3-SACT fulfills many of the desirable characteristics of an efficacy outcome measure. Although certainly less onerous than a CFA, consideration should be given to assessing whether the O3-SACT can exclude either the 12- or 24-hour time point and remain accurate since these time points add to participant burden. Given that the CFA is regarded as the accepted efficacy measure, future trials of PERT should include the O3-SACT measure for comparison to CFA results to further advance alternative, more feasible endpoints to assess PERT efficacy.

Fat soluble vitamin levels

Fat soluble vitamin (FSV) deficiency is common in EPI, but pancreatic enzyme supplementation often does not restore normal FSV levels. One or more FSV must be supplemented to achieve normal levels. One study of PERT (Zenpep) vs placebo used serum vitamin A and E levels as an outcome measure [26]. While levels of both vitamins increased on PERT vs placebo there was substantial overlap of the values. FSV levels are not suitable as primary outcome measures in clinical trials of PERT.

Measures of protein and carbohydrate

Although clinical use and efficacy studies of PERT focus on fat digestion and absorption, porcine PERT products also contain proteases and amylases which aid in digestion and absorption of protein and carbohydrates, respectively. The coefficient of nitrogen absorption (CNA) is often determined from the same stool collection used for the CFA measure and is often reported in PERT trials as a secondary outcome measure. Change in CNA tracks with CFA change in PERT trials, but not necessarily for individual subjects, including healthy volunteers and subjects with CF [7]. Other methods to assess protein digestion/absorption are under investigation and may hold promise as outcome measures in PERT trials, including continuous oral stable isotope ingestion to measure protein digestibility and whole-body protein turnover [27, 28] and a ninhydrin-based high-throughput method to detect all absorbed nitrogen in amine groups [29]. In the aforementioned study of the non-porcine PERT ANG003, protein digestion and absorption was evident using a novel O3-SACT that contained whey. Levels of absorbed free amino acids and peptides were higher with ANG003 protease compared to the off-PERT period [25].

Published reports of carbohydrate digestion and absorption are lacking from PERT trials, due in part to the intrinsic inability to test for carbohydrate that results from PERT in people with EPI, given the extra-pancreatic sources of amylase. Moreover, there is not a validated test available for research purposes. Whether continuous glucose monitoring (CGM) devices to calculate glucose AUC/excursions in response to a starch oral bolus is

effective in assessing carbohydrate absorption is under investigation [30]. In the ANG003 trial, a 4-hour CGM was performed during the O3-SACT (which contained potato starch), and revealed slightly lower glucose levels and insulin release with ANG003 amylase compared to the off-PERT period [25].

CLINICAL AND PATIENT REPORTED OUTCOME MEASURES

Clinical measures including body weight; stool weight, frequency and characteristics; and EPI-related symptoms like abdominal cramps and flatulence are commonly used as secondary efficacy measures in PERT studies, most notably during the supervised periods in a controlled setting while obtaining the primary outcome measure. Weight change, particularly observed during longer treatment periods, is most relevant to the pediatric population where growth charts are closely scrutinized, and appropriate weight gain is critical for development. Weight change may be relevant in underweight adults when it is attributed to severe EPI, but this may be multifactorial in the setting of pancreatic cancer or chronic pancreatitis which have additional implications on metabolism beyond EPI alone. Similarly, individual symptoms like stool consistency and fatty meal tolerance ought to improve with PERT, but these are subjective measures and can be affected by comorbid disorders of gut-brain interaction or learned behaviors related to post-prandial distress. Additionally, a placebo-controlled trial of pancrelipase (Creon) revealed a subset of subjects who reported no clinical signs or symptoms during the study [8]. Ideally, these subjective measures should be captured as part of a broader gastrointestinal symptom survey to capture gastrointestinal health with greater resolution. A participant's subjective experience of EPI and response to PERT must be considered as an outcome measure, but these symptoms are neither sensitive nor specific for diagnosis or monitoring of EPI, so these should not be considered as primary measures of efficacy for clinical trials of PERT [31].

Patient reported outcome measures (PROMs) for EPI include general gastroenterology PROMs which incorporate upper and lower gastrointestinal surveys, such as the Patient Assessment of Upper Gastrointestinal Disorders-Symptom Questionnaire (PAGI-SYM) [32] and the Patient Assessment of Constipation Symptom and Quality of Life Questionnaires (PAC-SYM and PAC-QOL) [33, 34] and symptom indices for a single disease, such as the Cystic Fibrosis Abdomen Score (CFAbd-Score) [35] or the Cystic Fibrosis Questionnaire-Revised (CFQ-R) [36, 37]. The CFAbd-Score has been used in several studies to assess the effect of CFTR modulator therapy on GI symptoms in pwCF, with the suggestion that the instrument meets FDA criteria for the development and validation of a PROM [38]. However, this tool is proprietary, centrally scored, and not universally available for independent use by investigators or

sponsors. The aforementioned PROMs each capture aspects of gastrointestinal health but fail to capture all aspects of the EPI experience, so additional questionnaires have been developed which are tailored more specifically to EPI. Among these latter PROMs is the Pancreatic Exocrine Insufficiency Questionnaire (PEI-Q) which was developed and validated for use in assessing EPI symptoms and their impact on health-related quality of life [39-41]. The PEI-Q includes 3 domains; 1) abdominal symptoms and 2) bowel symptoms which together are summed to the "symptom score" and 3) an impact on daily life domain which is designated the "impact score." Added together, the 3 domains provide a "total summary score." This has been used in two non-CF studies to date [42, 43] and while it provides a qualitative assessment of symptoms, it does not correlate with fecal elastase.

We discourage the use of non-validated or single-item PROMs as stand-alone secondary outcome measures and instead encourage a full-length EPI PROM to best capture the patient experience of EPI. At the present time, the PEI-Q seems to be the most complete PROM available and needs to be studied in pwCF, with the potential for using this PROM as a secondary measure of efficacy in CF studies of PERT.

REFERENCES

[1] A. Khan, S.S. Vege, V. Dudeja, S.T. Chari, Staging exocrine pancreatic dysfunction, Pancreatology 22(1) (2022) 168-172.

[2] U.R.R. Somaraju, A. Solis-Moya, Pancreatic enzyme replacement therapy for people with cystic fibrosis, Cochrane Database Syst Rev 8(8) (2020) CD008227.

[3] P. Chu, J. Mioc, P. O'Donovan, O. Henry, Clinical Efficacy and Safety of Treatments for Exocrine Pancreatic Insufficiency: A Systematic Literature Review, Digestion (2024) 1-17.

[4] M.W. Konstan, D. Borowitz, N. Mayer-Hamblett, C. Milla, L. Hendeles, S. Murray, R.A. Kronmal, S. Casey, L.M. Rose, W.J. Morgan, B.W. Ramsey, Study design considerations for evaluating the efficacy and safety of pancreatic enzyme replacement therapy in patients with cystic fibrosis, Clin Investig (Lond) 3(8) (2013) 731-741.

[5] R.C. Stern, J.D. Eisenberg, J.S. Wagener, R. Ahrens, M. Rock, G. doPico, D.M. Orenstein, A comparison of the efficacy and tolerance of pancrelipase and placebo in the treatment of steatorrhea in cystic fibrosis patients with clinical exocrine pancreatic insufficiency, Am J Gastroenterol 95(8) (2000) 1932-8.

[6] M.W. Konstan, R.C. Stern, J.R. Trout, J.M. Sherman, H. Eigen, J.S. Wagener, C. Duggan, M.E. Wohl, P. Colin, Ultrase MT12 and Ultrase MT20 in the treatment of exocrine pancreatic insufficiency in cystic fibrosis: safety and efficacy, Aliment Pharmacol Ther 20(11-12) (2004) 1365-71.

[7] D. Borowitz, M.W. Konstan, A. O'Rourke, M. Cohen, L. Hendeles, F.T. Murray, Coefficients of fat and nitrogen absorption in healthy subjects and individuals with cystic fibrosis, J Pediatr Pharmacol Ther 12(1) (2007) 47-52.

[8] B.C. Trapnell, K. Maguiness, G.R. Graff, D. Boyd, K. Beckmann, S. Caras, Efficacy and safety of Creon 24,000 in subjects with exocrine pancreatic insufficiency due to cystic fibrosis, J Cyst Fibros 8(6) (2009) 370-7.

[9] A.R. Opekun, Jr., F.M. Sutton, Jr., D.Y. Graham, Lack of dose-response with Pancrease MT for the treatment of exocrine pancreatic insufficiency in adults, Aliment Pharmacol Ther 11(5) (1997) 981-6.

[10] D. Borowitz, J.F. Brooks, E. Aliaj, D. Gallotto, Community Perspective on Pancreatic Enzyme Replacement Therapy in Cystic Fibrosis, J Pediatr Gastroenterol Nutr 75(5) (2022) e94-e97.

[11] S. Caras, D. Boyd, L. Zipfel, S. Sander-Struckmeier, Evaluation of stool collections to measure efficacy of PERT in subjects with exocrine pancreatic insufficiency, J Pediatr Gastroenterol Nutr 53(6) (2011) 634-40.

[12] D. Borowitz, N. Aronoff, L.C. Cummings, A. Maqbool, A.E. Mulberg, Coefficient of Fat Absorption to Measure the Efficacy of Pancreatic Enzyme Replacement Therapy in People With Cystic Fibrosis: Gold Standard or Coal Standard?, Pancreas 51(4) (2022) 310-318.

[13] S.D. Freedman, K. Zaworski, K. Pierzynowska, S. Pierzynowski, R. Gallotto, M. Sathe, D.S. Borowitz, Validation of an omega-3 substrate challenge absorption test as an indicator of global fat lipolysis, PLoS One 18(5) (2023) e0284651.

[14] W.Y. Gao, A.E. Mulberg, Dependence of PERT endpoint on endogenous lipase activity, Pancreas 43(8) (2014) 1232-8.

[15] J.C. Delchier, J.C. Soule, BT-PABA test with plasma PABA measurements: evaluation of sensitivity and specificity, Gut 24(4) (1983) 318-25.

[16] G.R. Vantrappen, P.J. Rutgeerts, Y.F. Ghoos, M.I. Hiele, Mixed triglyceride breath test: a noninvasive test of pancreatic lipase activity in the duodenum, Gastroenterology 96(4) (1989) 1126-34.

[17] J.E. Dominguez-Munoz, J. Iglesias-Garcia, M. Vilarino-Insua, M. Iglesias-Rey, 13C-mixed triglyceride breath test to assess oral enzyme substitution therapy in patients with chronic pancreatitis, Clin Gastroenterol Hepatol 5(4) (2007) 484-8.

[18] V.A. Stallings, J.T. Mondick, J.I. Schall, J.S. Barrett, M. Wilson, M.R. Mascarenhas, Diagnosing malabsorption with systemic lipid profiling: pharmacokinetics of pentadecanoic acid and triheptadecanoic acid following oral administration in healthy subjects and subjects with cystic fibrosis, Int J Clin Pharmacol Ther 51(4) (2013) 263-73.

[19] J.N. Brownell, J.I. Schall, V.A. Stallings, Pancreatic Function in Chronic Pancreatitis: A Cohort Study Comparing 3 Methods of Detecting Fat Malabsorption and the Impact of Short-term Pancreatic Enzyme Replacement Therapy, Pancreas 48(8) (2019) 1068-1078.

[20] M.R. Mascarenhas, J. Mondick, J.S. Barrett, M. Wilson, V.A. Stallings, J.I. Schall, Malabsorption blood test: Assessing fat absorption in patients with cystic fibrosis and pancreatic insufficiency, J Clin Pharmacol 55(8) (2015) 854-65.

[21] A.E. Phillips, J.N. Brownell, A. Tindall, B.D. Kiernan, D. Patel, D. Gelfond, V.A. Stallings, Proton-Pump Inhibitors and Fat Absorption in Cystic Fibrosis and Pancreatic Insufficiency: A Randomized Crossover Pilot Trial, Dig Dis Sci (2024).

[22] J. Stevens, C. Wyatt, P. Brown, D. Patel, D. Grujic, S.D. Freedman, Absorption and Safety With Sustained Use of RELiZORB Evaluation (ASSURE) Study in Patients With Cystic Fibrosis Receiving Enteral Feeding, J Pediatr Gastroenterol Nutr 67(4) (2018) 527-532.

[23] S. Freedman, D. Orenstein, P. Black, P. Brown, K. McCoy, J. Stevens, D. Grujic, R. Clayton, Increased Fat Absorption From Enteral Formula Through an In-line Digestive Cartridge in Patients With Cystic Fibrosis, J Pediatr Gastroenterol Nutr 65(1) (2017) 97-101.

[24] K. Goncharova, Enhanced absorption of long-chain polyunsaturated fatty acids following consumption of functional milk formula, pre-digested with immobilized lipase ex vivo, in an exocrine pancreatic insufficient (EPI) pig model, Journal of Functional Foods 34(July 2017) (2017) 422-430.

[25] M. Sathe, S.D. Freedman, M.S. Putman, R. Gallotto, M. Clarkin, D. Gallotto, K. Pierzynowska, D. Borowitz, Results of a clinical trial of ANG003, a non-porcine pancreatic enzyme replacement therapy, in people with cystic fibrosis, J Cyst Fibros https://doi.org/10.1016/j.jcf.2025.07.008 (2025).

[26] J.L. Wooldridge, J.E. Heubi, R. Amaro-Galvez, S.R. Boas, K.V. Blake, S.Z. Nasr, B. Chatfield, S.A. McColley, M.S. Woo, K.A. Hardy, R.M. Kravitz, C. Straforini, M. Anelli, C. Lee, EUR-1008 pancreatic enzyme replacement is safe and effective in patients with cystic fibrosis and pancreatic insufficiency, J Cyst Fibros 8(6) (2009) 405-17.

[27] M.P. Engelen, G. Com, P.J. Anderson, N.E. Deutz, New stable isotope method to measure protein digestibility and response to pancreatic enzyme intake in cystic fibrosis, Clin Nutr 33(6) (2014) 1024-32.

[28] K.R. Hirsch, D.D. Church, R.R. Wolfe, A.A. Ferrando, Continuous oral stable isotope ingestion to measure whole-body protein turnover, Clin Nutr ESPEN 49 (2022) 385-389.

[29] K. Pierzynowska, K. Zaworski, P. Wychowanski, J. Donaldson, J. Wolinski, D. Borowitz, R. Gallotto, S. Pierzynowski, Modified throughput ninhydrin method for the qualitative assessment of dietary protein absorption in pig plasma, Biol Methods Protoc 9(1) (2024) bpae078.

[30] M. Uemura, Y. Yano, T. Suzuki, T. Yasuma, T. Sato, A. Morimoto, S. Hosoya, C. Suminaka, H. Nakajima, E.C. Gabazza, Y. Takei, Comparison of Glucose Area Under the Curve Measured Using Minimally Invasive Interstitial Fluid Extraction Technology with Continuous Glucose Monitoring System in Diabetic Patients, Diabetes Metab J 41(4) (2017) 265-274.

[31] D.C. Whitcomb, A.M. Buchner, C.E. Forsmark, AGA Clinical Practice Update on the Epidemiology, Evaluation, and Management of Exocrine Pancreatic Insufficiency: Expert Review, Gastroenterology 165(5) (2023) 1292-1301.

[32] A.M. Rentz, P. Kahrilas, V. Stanghellini, J. Tack, N.J. Talley, C. de la Loge, E. Trudeau, D. Dubois, D.A. Revicki, Development and psychometric evaluation of the patient assessment of upper gastrointestinal symptom severity index (PAGI-SYM) in patients with upper gastrointestinal disorders, Qual Life Res 13(10) (2004) 1737-49.

[33] Y. Yiannakou, J. Tack, H. Piessevaux, D. Dubois, E.M.M. Quigley, M.Y. Ke, S. Da Silva, A. Joseph, R. Kerstens, The PAC-SYM questionnaire for chronic constipation: defining the minimal important difference, Aliment Pharmacol Ther 46(11-12) (2017) 1103-1111.

[34] P. Marquis, C. De La Loge, D. Dubois, A. McDermott, O. Chassany, Development and validation of the Patient Assessment of Constipation Quality of Life questionnaire, Scand J Gastroenterol 40(5) (2005) 540-51.

[35] H. Tabori, A. Barucha, C. Zagoya, F. Duckstein, G.A. Dunay, P. Sadrieh, L. Polte, J.G. Mainz, Scoring Abdominal Symptoms in People with Cystic Fibrosis, J Clin Med 13(6) (2024).

[36] A.J. Freeman, M. Sathe, E. Aliaj, D. Borowitz, B. Fogarty, C.H. Goss, S. Freedman, S.L. Heltshe, U. Khan, D. Riva, C. Roman, M. Romasco, S.J. Schwarzenberg, C.A. Ufret-Vincenty, B. Moshiree, Designing the GALAXY study: Partnering with the cystic fibrosis community to optimize assessment of gastrointestinal symptoms, J Cyst Fibros 20(4) (2021) 598-604.

[37] M. Lee, M. Sathe, B. Moshiree, P.T. Vu, S.L. Heltshe, S.J. Schwarzenberg, S.D. Freedman, A.J. Freeman, Estimating minimal clinically important difference (MCID) for gastrointestinal symptoms in cystic fibrosis, J Cyst Fibros 23(5) (2024) 991-999.

[38] J.G. Mainz, K. Lester, B. Elnazir, M. Williamson, E. McKone, D. Cox, B. Linnane, C. Zagoya, F. Duckstein, A. Barucha, J.C. Davies, P. McNally, R.S. Group, Reduction in abdominal symptoms (CFAbd-Score), faecal M2-pyruvate-kinase and Calprotectin over one year of treatment with Elexacaftor-Tezacaftor-Ivacaftor in people with CF aged >/=12 years - The RECOVER study, J Cyst Fibros 23(3) (2024) 474-480.

[39] C.D. Johnson, R. Arbuckle, N. Bonner, G. Connett, E. Dominguez-Munoz, P. Levy, D. Staab, N. Williamson, M.M. Lerch, Qualitative Assessment of the Symptoms and Impact of Pancreatic Exocrine Insufficiency (PEI) to Inform the Development of a Patient-Reported Outcome (PRO) Instrument, Patient 10(5) (2017) 615-628.

[40] C.D. Johnson, N. Williamson, G. Janssen-van Solingen, R. Arbuckle, C. Johnson, S. Simpson, D. Staab, E. Dominguez-Munoz, P. Levy, G. Connett, M.M. Lerch, Psychometric evaluation of a patient-reported outcome measure in pancreatic exocrine insufficiency (PEI), Pancreatology 19(1) (2019) 182-190.

[41] Abbott. Pancreatic Exocrine Insufficiency Questionnaire https://acare.abbott.com/en/digestive-health/pei-q/. (Accessed 24 March 2025).

[42] J.M. Lee, S.H. Lee, Y.H. Choi, S.Y. Han, J.H. Jo, J.W. Choe, E.J. Kim, D.K. Jang, M.K. Jung, Association between severity of pancreatic exocrine insufficiency and computed tomography-based morphological severity in patients with chronic pancreatitis, Medicine (Baltimore) 103(48) (2024) e40737.

[43] V. Parihar, R. Ballester, P.F. Ridgway, K.C. Conlon, J. Gibney, B.M. Ryan, Screening for undiagnosed pancreatic exocrine insufficiency (PEI) in a cohort of diabetic patients using faecal elastase testing and PEI scoring system, Acta Diabetol 61(10) (2024) 1301-1307.
